# A study on the causal relationship between the gut microbiome and herpes zoster using Mendelian randomization

**DOI:** 10.3389/fmed.2024.1442750

**Published:** 2024-08-30

**Authors:** Zenan Meng, Tingting Wang, Yue Liao, Xinzhi Li

**Affiliations:** ^1^Hubei Key Laboratory of Tumor Microenvironment and Immunotherapy, College of Basic Medical Sciences, China Three Gorges University, Yichang, China; ^2^Shaoxing Yuecheng District People's Hospital, Shaoxing, China; ^3^College of Medicine and Health Sciences, China Three Gorges University, Yichang, China; ^4^Affiliated Renhe Hospital of China Three Gorges University, Yichang, China

**Keywords:** herpes zoster, gut microbiome, dermatology, Mendelian randomized grouping, pathogenic factors

## Abstract

**Introduction:**

The relationship between herpes zoster recurrence and the gut microbiome was not studied. We analyzed data on the gut microbiome and herpes zoster from the Large-Scale Genome-Wide Association Study (GWAS) database using bidirectional Mendelian randomization. For the first time, we identified a potentially bidirectional causal relationship between the gut microbiome and herpes zoster (HZ). These findings are groundbreaking and hold promise for new directions in the treatment of HZ, a global disease.

**Background and aims:**

HZ had a high global incidence, characterized by shingled blisters, blood blisters, and neuropathic pain, and could develop in various parts of the body, including the ear and throat. It was believed its onset was closely related to old age and infirmity. Some studies reported that the incidence of herpes zoster in patients with inflammatory intestinal diseases (such as Crohn’s disease and ulcerative colitis) was higher than in the general population. Existing studies attributed this to the reactivation of varicella-zoster virus (VZV) due to autoinflammatory attacks and immunosuppressive drugs. This provided a basis for exploring the new pathogenesis of HZ and investigating whether there was a relationship between intestinal auto-flora and the development of HZ. This study aimed to examine this potential relationship using bidirectional Mendelian analyses.

**Methods:**

GWAS data on HZ and gut microbiota were obtained from FinnGen, the Mibiogen consortium, and HZ meta-analysis data from the IEU Open GWAS Project. These data were subjected to two-sample Mendelian randomization (MR) analysis to determine if there is a causal relationship between gut microbiota and HZ. Additionally, bidirectional Mendelian analyses were conducted to identify the direction of causality and to clarify any potential interactions.

**Results:**

In our Mendelian Randomization (MR) analysis, we identified, for the first time, two gut microbes that might be associated with HZ reactivation. In the reverse MR analysis, four gut microbiota showed a potential association between the genetic susceptibility of gut microbiota and HZ reactivation. We found that genus Tyzzerella3 (OR: 1.42, 95% CI: 1.17–1.72, FDR < 0.1) may be strongly correlated with an increased probability of HZ (ICD-10: B02.901) reactivation. Additionally, phylum Cyanobacteria was identified as a potential risk factor for the onset of HZ rekindling (OR: 1.42, 95% CI: 1.09–1.87). Analyzing the results of the reverse MR, we also identified a potential inhibitory effect (OR: 0.91, 95% CI: 0.84–0.99) of HZ onset on the genus Eubacteriumhallii group in the gut, suggesting that HZ might reduce its abundance. However, genus Escherichia/Shigella (OR: 1.11, 95% CI: 1.01–1.22), genus Veillonella (OR: 1.16, 95% CI: 1.04–1.30), and phylum Proteobacteria (OR: 1.09, 95% CI: 1.01–1.18) appeared to act as potential protective factors, indicating that the relative abundance and viability of these three bacteria increased in the HZ state.

**Conclusion:**

We identified the influence of gut flora as a new causative factor for HZ reactivation. Additionally, we found that individuals suffering from HZ might potentially impact their gut flora. Specific bacterial taxa that could influence the onset and progression of HZ were identified, potentially providing new directions for HZ treatment.

## 1 Introduction

Herpes zoster (HZ) is an acute skin infection caused by the reactivation of the varicella-zoster virus (VZV). It is clinically characterized by pain, itching, and a rash, usually confined to a single ganglion in a region of skin distribution. Although HZ is more common in the elderly and immunocompromised individuals, its incidence has also increased in younger people in recent years. Reactivation of HZ causes great discomfort and a decreased quality of life for patients, especially in cases of severe complications, such as intense pain along the infected nerve course (1).

The gut microbiome, as an important ecosystem in the human body, significantly impacts the host’s immune system, metabolism, and nervous system. Recent studies have shown that imbalances in the gut microbiome are strongly associated with the onset and progression of various diseases, including skin diseases (2). However, regarding the relationship between the gut microbiome and HZ, only six genera reported by Deng et al. are potentially relevant (3). Further systematic studies are needed due to the complexity of the regulatory mechanisms of the intestinal flora, the pathogenic mechanisms of HZ, and the variety of potential confounding factors.

In this context, the design of Mendelian randomization (MR) studies of the gut microbiome becomes particularly important. The MR research design is a methodology capable of determining causality and assessing the impact of interventions on outcomes by using instrumental variables (IVs) closely related to the exposure. This method randomly assigns subjects to experimental and control groups to study causality and address confounders (4–6). Therefore, some previous studies have explored the causal relationship between HZ disease and primary membranous nephropathy using MR methods (7). Zou et al. also reported a potential association between Crohn’s disease (CD) in inflammatory bowel disease (IBD) and chickenpox in VZV infections through MR analysis (8). Thus, when studying the causal relationship between the gut microbiome and HZ, MR design methods have the potential to analyze the association with greater accuracy.

In conclusion, this study aimed to systematically assess the causal relationship between the gut microbiome and HZ through a two-way, two-sample Mendelian randomization analysis. We expect this study to provide a deeper understanding of the impact of the gut microbiome on the pathogenesis of HZ and offer new ideas and strategies for its prevention and treatment.

## 2 Research design and methodology

### 2.1 Study design

**Figure 1:**
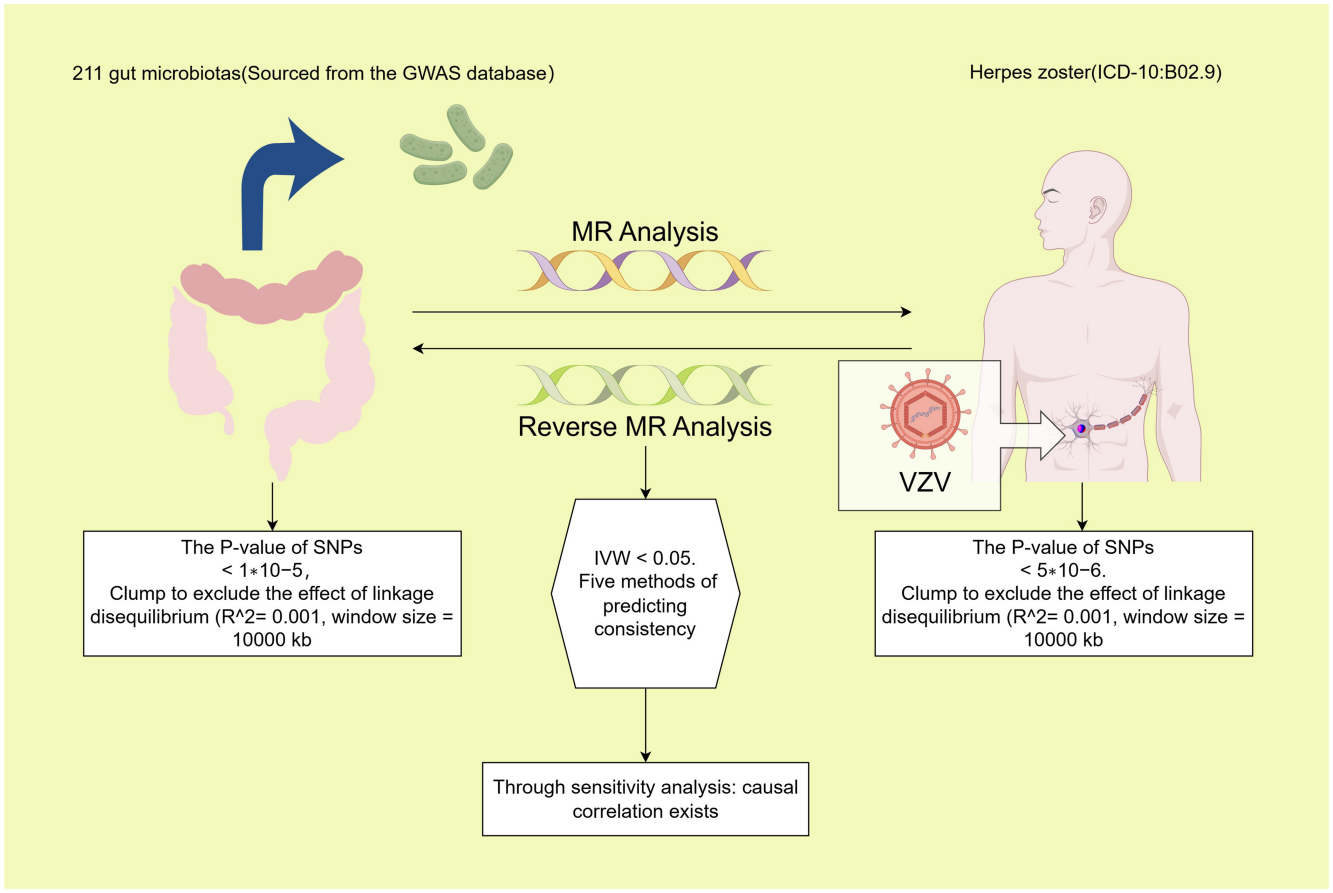
Process diagram for the causal analysis of gut microbiome and herpes zoster. Data from 211 gut microbiome were efficiently processed to select instrumental variables that met the requirements: (1) *p*-value <1 × 10^–5; (2) SNPs within a window size of 10,000 kb were pruned at a threshold of r^2 < 0.001 to mitigate linkage disequilibrium (LD). After harmonization, MR analysis was performed using five methods. A bacterial colony was considered significant when the IVW *p*-value was <0.05 and the estimates from the five analysis methods were consistent. Significant colonies were then tested for pleiotropy and heterogeneity. Polyvalent and heterogeneous flora were discarded. Significant bacterial flora were screened out. Then, reverse MR analysis was performed with exposure and outcome positions swapped and a *p*-value <5 × 10^–6; the rest of the analysis was consistent with the initial MR analysis.

In this investigation, 211 gut microbiota were designated as exposures, with HZ chosen as the endpoint for MR analysis. The same dataset was utilized concurrently, with the positions of exposure and outcome interchanged for reverse MR analysis. All MR analyses in the sub-study were conducted based on three fundamental assumptions: (1) Instrumental Variables (IVs) must exhibit a strong correlation with exposure, (2) IVs should not be correlated with confounders, and (3) IVs can solely influence the outcome through exposure factors (9). The study’s schematic is presented in Figure 1.

### 2.2 Gut microbiome GWAS data

The large-scale GWAS pooled data of the gut microbiome from the Mibiogen consortium covers a diverse range of microbial species and genetic variants, ensuring a comprehensive reflection of gut microbiota diversity. It boasts one of the largest sample sizes among available studies, thus ensuring reliable statistical analyses and sufficient statistical power. The dataset can be downloaded from: https://mibiogen.gcc.rug.nl/. It included data from 18,340 individuals across 24 cohorts. In this study, we analyzed gut microbiota composition using three different regions of the 16S rRNA gene and identified genetic variations influencing the relative abundance of microbial taxa through mapping microbiota quantitative trait loci (mbQTL), all within the GWAS database. To standardize our analysis, all datasets were normalized to 10,000 reads per sample to adjust for sequencing depth variations. Finally, after excluding unknown gut microbiota, we finalized 211 taxa, comprising 35 families, 20 orders, 16 phyla, 9 classes, and 131 genera, for inclusion in the Mendelian randomization (MR) analysis using GWAS summary statistics (10).

### 2.3 .HZ GWAS data

The International Classification of Diseases-10 (ICD-10) diagnosis code for HZ is B02.9, and GWAS summary data for HZ (B02.9) are sourced from the FinnGen database. This database draws its data from Finland’s National Health Record System and Biospecimen Bank, encompassing diverse populations and extensive clinical data. The FinnGen dataset undergoes rigorous quality control procedures, including genotyping quality assurance, mismatch detection, sample cleaning, and data standardization, ensuring the accuracy and reliability of the data. Detailed medical records and diagnostic information further validate the precision of the case definition. Accessed via download from GWAS.^1^ The dataset is named finn-b-AB1_ZOSTER and consists of a European population, including both males and females. It comprises 2080 cases and 211,856 controls for HZ (B02.9), with a total of 16,380,433 SNPs. All individuals included in the study were of European origin (11).

### 2.4 Selection of IVs

IVs play a crucial role in MR studies. The selection of appropriate IVs must adhere to three essential criteria: (1) Relevance: IVs must exhibit a strong correlation with exposure factors, such as characteristics of the gut microbiome. Genetic variants (SNPs) significantly associated with exposure are typically identified through GWAS, where these SNPs explain a substantial portion of the variation in the exposure factors. (2) Independence: IVs should be independent of potential confounders. This necessitates that these genetic variants are not directly associated with any confounding factors other than the exposure (e.g., age, sex, lifestyle), a criterion validated through statistical modeling and biological knowledge. (3) Exclusion Restriction: An IV should influence the outcome (e.g., herpes zoster) solely through the exposure factor and not through any other pathways. While challenging to fully confirm in practice, methods like the MR-Egger intercept are employed to assess and adjust for potential violations of this assumption, particularly addressing horizontal pleiotropy (12, 13). Therefore, adhering to the above three principles, we selected SNPs that were statistically significant as IVs for the study. The criteria for selecting IVs were as follows: (1) SNPs significantly associated with 211 gut microbiota were selected as potentially eligible IVs using the TwoSampleMR package of the R software (SNP *p*-value <1 × 10^−5) (14); (2) SNPs were clustered to exclude the effect of linkage disequilibrium (R^2 = 0.001, clumping distance = 10,000 kb) to ensure the independence of IVs (15); (3) palindromic alleles were removed, and SNPs with the smallest *p*-values related to traits were retained, resulting in a total of 211 bacterial traits. For the reverse MR analysis, HZ IVs were filtered using the same criteria. Unfortunately, selecting *p* ≤ 5 × 10^−8 would have filtered out too many small SNPs, leaving no conforming SNPs. Therefore, we chose *p* ≤ 5 × 10^−6 as the criterion and adjusted the cluster (R^2 = 0.001, window size = 10,000 kb) (16–19). Other criteria remained the same. To avoid weak instrumental bias, the F-statistic for each bacterial taxon was calculated as follows.


$$F=R^{2}\times \frac{N-1-K}{\left(1-R^{2}\right)\times K}$$

In the formula, R^2 is used to explain the exposure variance of IVs, n is the sample size, and k is the number of IVs. Based on previous studies, an F statistic ≥10 was considered to be free of weak instrumental bias (20).

### 2.5 MR analysis

In this study, we employed five methods, namely IVW, MR Egger’s method, Weighted Median (WM), Simple Mode, and Weighted Mode, to estimate the causal effect of gut microbiota on HZ (21). Here, we focused on the properties of IVW, one of the most commonly used methods in Mendelian randomization analyses. IVW uses inverse variance weighting to obtain an estimate of causality by combining multiple single genotype–phenotype effect estimates. This method assumed that all genotypes had a homogeneous effect on the outcome and no level of impurity. Its simplicity made it easy to implement and usually statistically efficient. However, IVW had the disadvantage of being susceptible to instrumental variable multiplicity and heterogeneity when all instrumental variables were assumed valid. In such cases, more robust methods like the MR Egger method, which can handle possible levels of impurity or skewness in the causal estimates and provide consistent causal estimates, or the WM method, which combines the strengths of IVW and MR Egger and provides relatively robust causal estimates in the presence of skewness or impurity, were particularly important to improve the accuracy and robustness of the estimates (22, 23). In the absence of these effects, IVW was considered the most accurate method, even though the other four methods did not produce positive results. Therefore, we analyzed the relationship between gut microbiota and HZ primarily using the IVW method, supplemented by the MR Egger method, the WM method, the Simple Mode method, and the Weighted Mode method, to provide the most accurate estimates of the effects in this study. It was summarized that the results of MR analysis were mainly based on IVW, with the other four methods serving as auxiliary references to IVW, and a significant difference between HZ and gut microbiota was considered to exist when the estimates of the five methods were consistent and IVW < 0.05 (24). A significant difference between HZ and gut microbiota was considered to exist when the estimates of the five methods were consistent and IVW < 0.05. Additionally, after matching the IVs, we adopted the practice of excluding the matched group if the SNP count in the group was <3, as the statistical significance of the results in these groups was often unreliable due to the small number of SNPs.

### 2.6 Reverse MR analysis

In order to investigate whether HZ has a causal effect on other gut microbiota, we also conducted reverse MR analysis using SNPs strongly associated with HZ as instrumental variables (HZ as exposure and gut microbiota as outcome). The analysis procedure was similar to that of the MR analysis (6).

### 2.7 Analysis of horizontal pleiotropy and heterogeneity

Important microbiota were tested for pleiotropy and heterogeneity to ensure the accuracy of IVW results. The MR-Egger intercept test and Mendelian Randomization Pleiotropy RESidual Sum and Outlier (MR-PRESSO) global test were used to detect horizontal pleiotropy. The intercept term of MR-Egger regression provided a quantitative indicator for assessing horizontal pleiotropy in the instrumental variables. If the intercept term was not significantly different from zero, it indicated that the presence of horizontal pleiotropy was likely to be very small, thereby enhancing the reliability of IVW. MR-PRESSO is a global test for overall horizontal pleiotropy, residuals, and outliers, assessing the overall horizontal pleiotropy of the IVs and identifying anomalous SNPs that lead to pleiotropy (25). In short, both methods passing with a *p*-value >0.05 indicated that horizontal pleiotropy did not exist, in line with the principle of exclusivity (26). To assess the degree of heterogeneity, Cochran’s Q test was used, with *p*-value >0.05 considered non-heterogeneous. According to the literature, in the presence of heterogeneity (*p*-value <0.05), the inverse variance weighting (IVW) method uses a random effects model. Conversely, in the absence of heterogeneity, a fixed effects model is used. After implementing the random effects model, if heterogeneity persisted, the corresponding microbiota were excluded from the analysis (27). However, in the absence of observed heterogeneity, the results of the random effects model and the fixed effects model in IVW produced consistent results. Therefore, in our study, we calculated *p*-values using the random effects model in the IVW approach. This nuanced approach enhanced the robustness of the results obtained through the IVW method. Additionally, leave-one-out analysis was employed to exclude the effect of individual SNPs (28, 29).

### 2.8 .Data processing

All data processing and analysis were performed using R software (version 4.2.3).^2^ The R packages utilized in the study included TwoSampleMR, Mendelian Randomization, and MR-PRESSO (30).

## 3 Results

### 3.1 IVs for gut microbiome and HZ

The *p*-values for the gut flora-related SNPs in Supplementary Table S3 column (pval.outcome) ranged from 0.00052 to 0.9999, all of which were greater than 1 × 10^−5. Similarly, the *p*-values of the SNPs associated with herpes zoster in the column of Supplementary Table S4 (pval.outcome) ranged from 0.00022 to 0.99972, all of which were greater than 5 × 10^−6. Therefore, the IVs do not strongly correlate with the outcome and do not violate Hypothesis 3. The instrumental variables were then screened for strong correlation (*p*-value <1 × 10^−5) and clustered (r2 = 0.001, window size = 10,000 kb), resulting in 2,816 instrumental variables being extracted from the gut microbiome (Supplementary Table S1). For inverse MR analysis, HZ (B02.9) screened for IVs based on *p*-value <5 × 10^−6 and clumping (r2 = 0.001, window size = 10,000 kb), and finally 14 IVs (e.g., rs753064, rs4451553, rs79408779) were screened for HZ (B02.9) (Supplementary Table S1).

### 3.2 MR analysis of the causal impact of the gut microbiome on HZ risk

**Figure 2:**
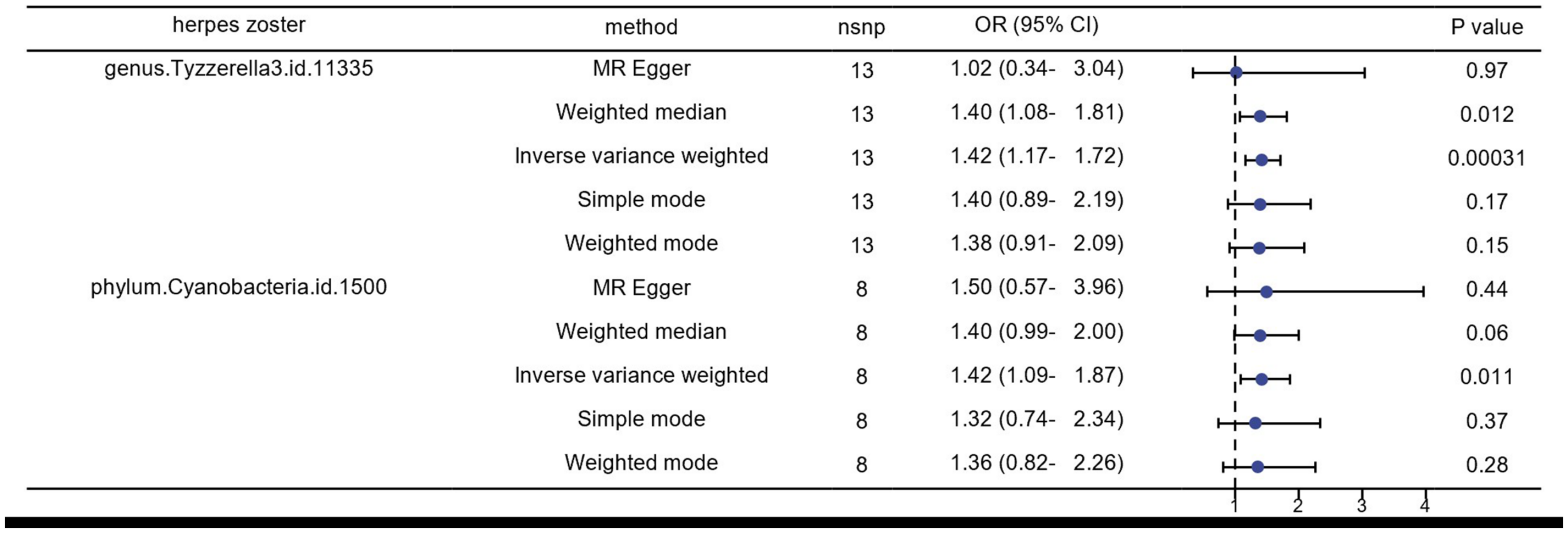
Results of MR analysis of the gut microbiome of patients with herpes zoster by five methods.

We identified several gut microorganisms with potential causal effects on HZ risk, as follows: genus.Tyzzerella3 (OR: 1.42, 95% CI: 1.17–1.72 FDR < 0.1) may strongly correlate with an increased probability of HZ (ICD-10: B02.901) reactivation. Additionally, phylum.Cyanobacteria is a potential risk factor for HZ rekindling (OR: 1.42, 95% CI: 1.09–1.87) (Figures 2, 4).

### 3.3 Causal effects of HZ on the gut microbiome: reverse MR analysis

**Figure 3:**
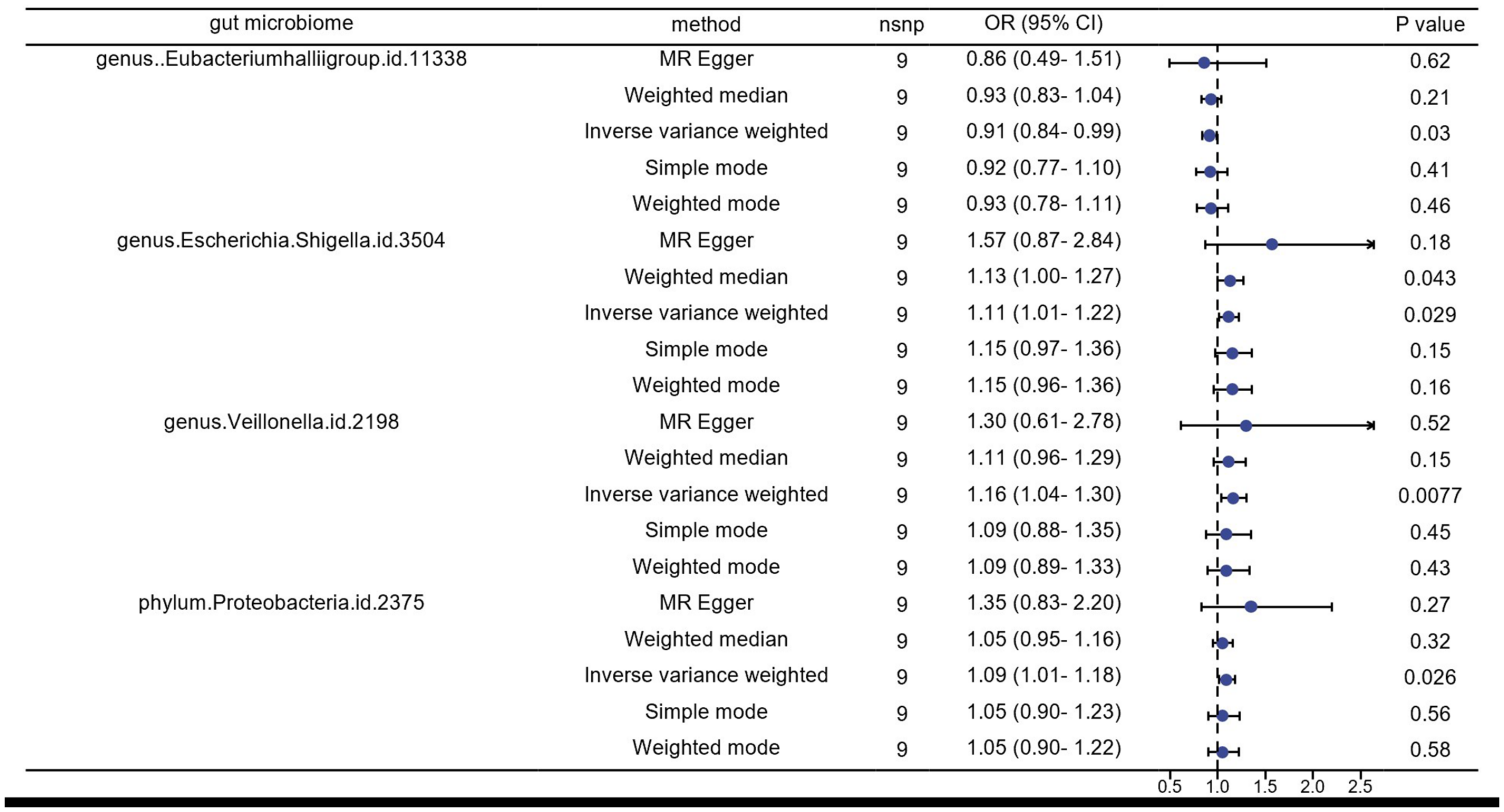
Results of inverse MR analysis of herpes zoster pathogenesis and the gut microbiome by five methods.

**Figure 4:**
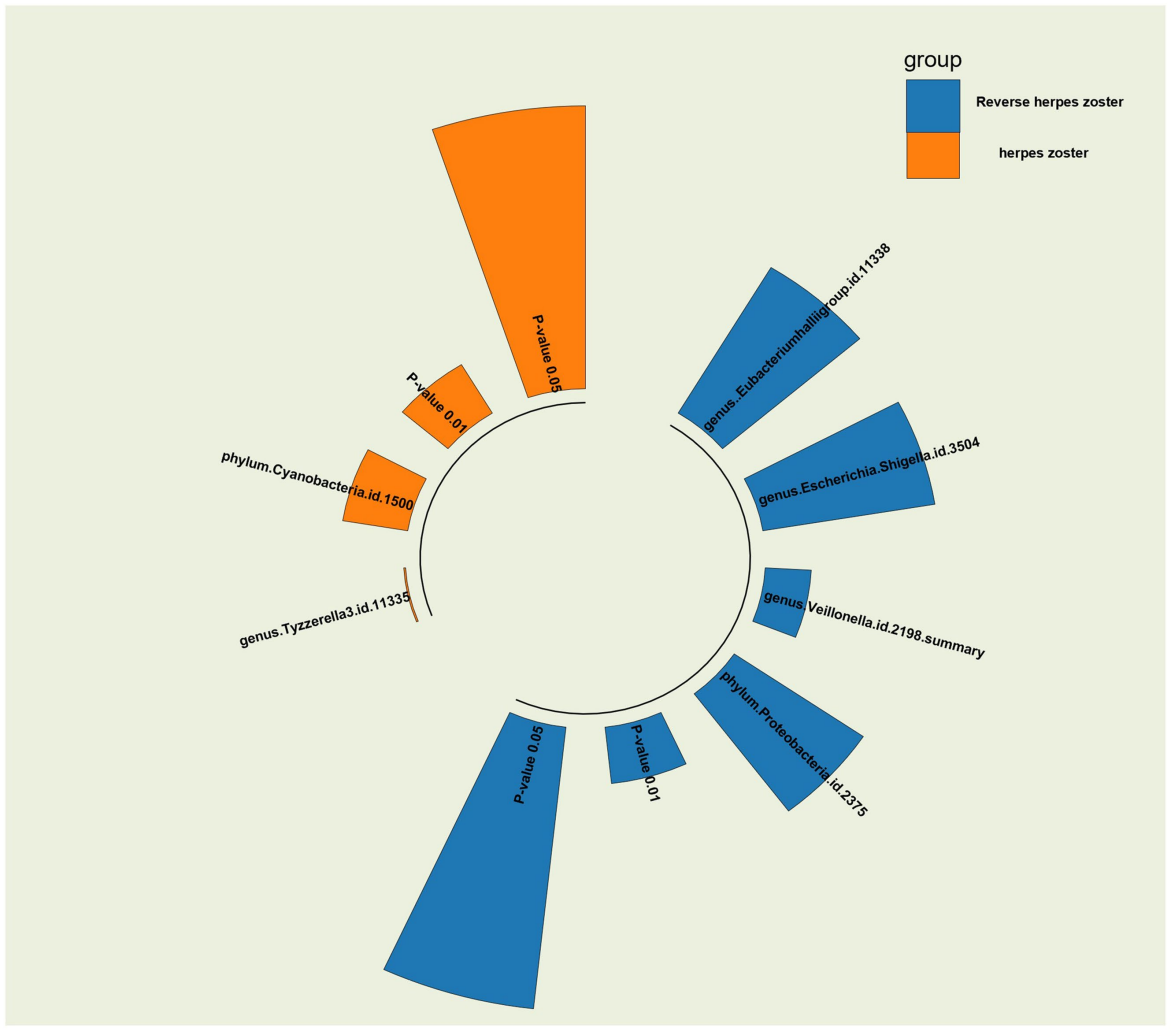
*p*-value radiographs of the results in MR and inverse MR analyses of the gut microbiome and herpes zoster for the IVW method are shown and visualized against *p*-value = 0.05 versus 0.01.

In the reverse Mendelian randomization analysis, we also observed a potential inhibitory effect of herpes zoster (HZ) onset on the genus Eubacteriumhallii group in the intestine (OR: 0.91, 95% CI: 0.84–0.99), indicating that HZ could be a risk factor leading to a decrease in its abundance. Conversely, genus Escherichia/Shigella (OR: 1.11, 95% CI: 1.01–1.22), genus Veillonella (OR: 1.16, 95% CI: 1.04–1.30), and phylum Proteobacteria (OR: 1.09, 95% CI: 1.01–1.18) emerged as potential protective factors, suggesting an increase in the relative abundance and viability of these three bacteria in the presence of HZ (Figures 3, 4).

### 3.4 MR sensitivity analysis

**Figure 5:**
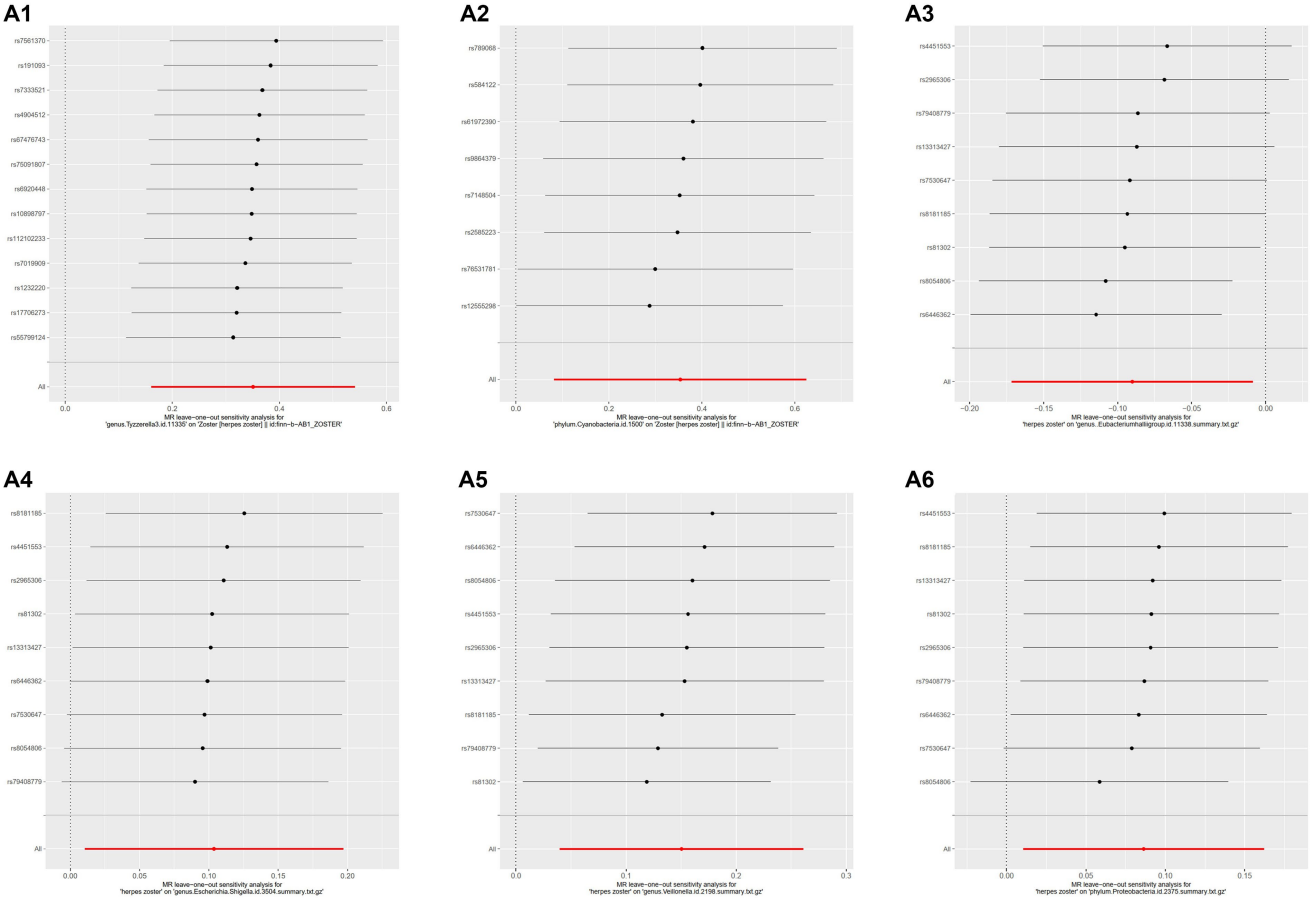
Results of leave-one-out analysis of several gut microbiota. MR analysis: **A1**, genus.Tyzzerella3; **A2**, phylum.Cyanobacteria. Reverse MR analysis: **A3**, genus.Eubacteriumhallii group; **A4**, genus.Escherichia.Shigella; **A5**, genus.Veillonella; **A6**, phylum.Proteobacteria.

**Figure 6:**
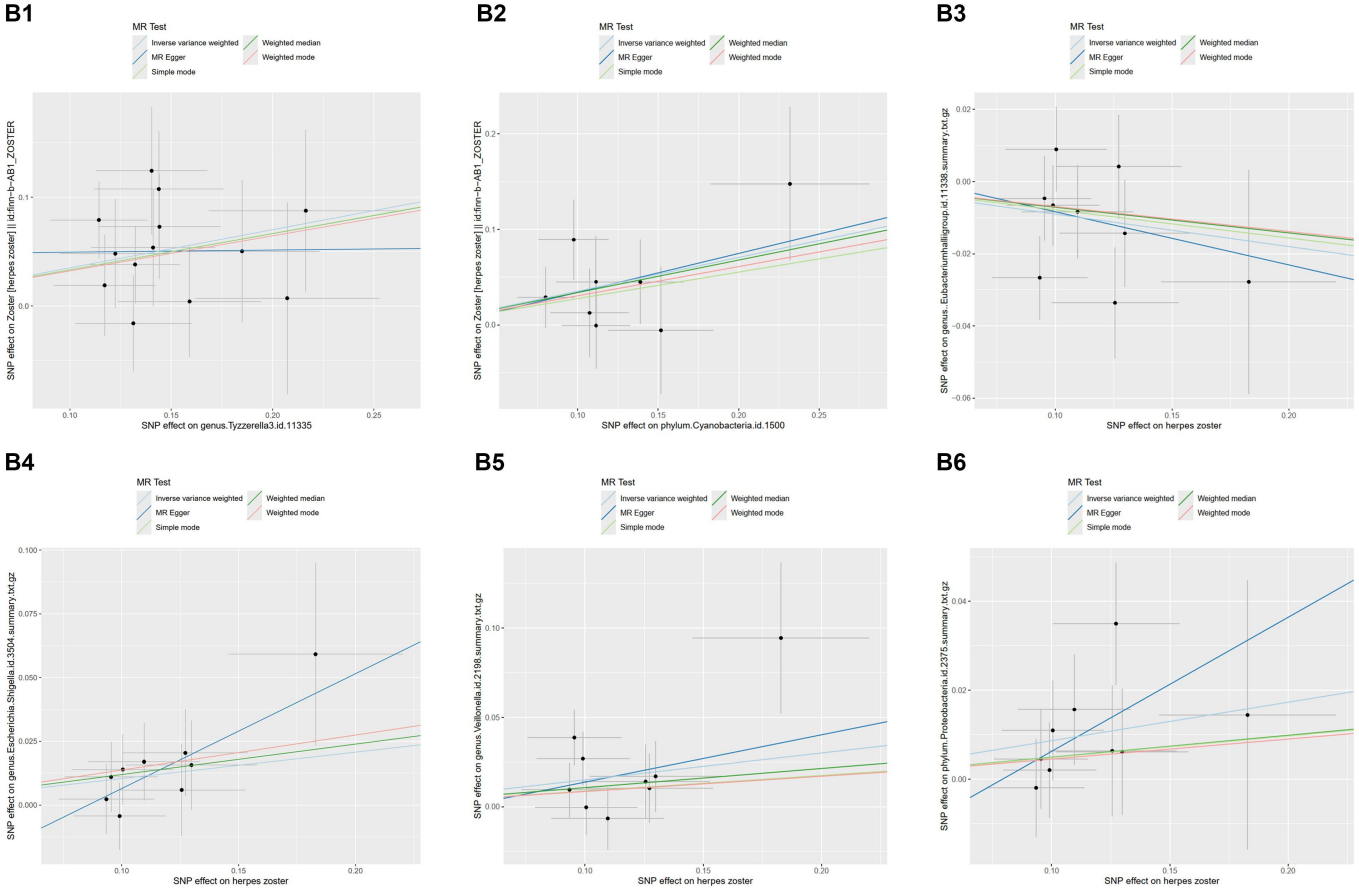
Scatterplots of the results of five methods of MR and reverse MR of several gut microbiota with herpes zoster.MR analysis: **B1**, genus.Tyzzerella3; **B2**, phylum.Cyanobacteria. Reverse MR analysis **B3**, genus.Eubacteriumhallii group; **B4**, genus.Escherichia.Shigella; **B5**, genus.Veillonella; **B6**, phylum.Proteobacteria. **B4**, genus.Escherichia.Shigella; **B5**, genus.Veillonella; **B6**, phylum.Proteobacteria.

MR-PRESSO did not detect significant zonal heterogeneity (Tables 1, 2), and Cochran’s Q test *p*-values indicated no heterogeneity in the study (Tables 1, 2) (*p*-values >0.05 for both tests). Furthermore, Leave-one-out analyses revealed no significantly different SNPs (Figure 5). MR-Egger regression showed no evidence of horizontal pleiotropy (*p*-value >0.05), and all F-statistic values exceeded 10. All analyses, including heterogeneity and sensitivity analyses, met the following criteria: IVW < 0.05, and estimates were consistent across all five methods (Figure 6).

**Table 1:** Multiplicity and heterogeneity of results from MR analysis.

Outcome	Exposure	Method	nsnp	MR-PRESSO global test	MR egger intercept test	Cochran’s Q test
Herpes zoster	genus.Tyzzerella3.id.11335	Inverse variance weighted	13	0.816	0.557857915	0.772164434
Herpes zoster	phylum.Cyanobacteria.id.1500	Inverse variance weighted	8	0.786	0.914737218	0.744842394

**Table 2:** Multiplicity and heterogeneity results from inverse MR analysis.

Outcome	Exposure	Method	nsnp	MR-PRESSO global test	MR egger intercept test	Cochran’s Q test
genus.Eubacteriumhalliigroup.id.11338	herpes zoster	Inverse variance weighted	9	0.423	0.844101061	0.393054924
genus.Escherichia.Shigella.id.3504	herpes zoster	Inverse variance weighted	9	0.916	0.285181117	0.911507311
genus.Veillonella.id.2198	herpes zoster	Inverse variance weighted	9	0.426	0.773464943	0.371864805
phylum.Proteobacteria.id.2375	herpes zoster	Inverse variance weighted	9	0.772	0.412703438	0.770511453

## 4 Discussion

In this study, we analyzed the potential causal relationship between 211 gut microbiota and HZ using two-sample MR analysis, as no previous relational analysis of the two had been conducted. Our study revealed, for the first time, a strong association between genus Tyzzerella3 and HZ development, along with a possible association involving phylum Cyanobacteria, both identified as risk factors for HZ. These findings suggest new targets for interventions aimed at modulating Tyzzerella3 to mitigate herpes zoster potentially. Additionally, we explored the impact of HZ pathogenesis on intestinal flora; for instance, we found that genus *Eubacterium hallii* group may be adversely affected under HZ conditions, potentially promoting the growth of genus Escherichia/Shigella, genus Veillonella, and phylum Proteobacteria. We further explored possible mechanisms: Tyzzerella 3, relatively understudied in the gut microbiome, is associated with metabolic and inflammatory diseases such as obesity, diabetes, arterial damage, and erectile dysfunction. This bacterium may modulate the systemic immune response and intestinal barrier function, potentially facilitating VZV reactivation. Its association with metabolic disorders and extraintestinal diseases suggests a role in weakening host immune defenses and increasing susceptibility to infections or latent virus activation (31, 32). Cyanobacteria, known to produce neurotoxins, may directly damage nerve cells, creating a conducive environment for VZV reactivation in the nervous system. Their metabolites could exacerbate nerve cell damage and inflammation, affecting immune responses systemically and in the gut, thereby influencing susceptibility to VZV reactivation (33).

The *Eubacterium hallii* group is a significant producer of Short-Chain Fatty Acids (SCFAs), including butyric acid and propionic acid, which play crucial roles in maintaining intestinal health and modulating the immune system. These SCFAs exert anti-inflammatory and immunomodulatory effects, supporting intestinal barrier integrity and reducing inflammatory responses. Given their susceptibility to immune disorders, increased intestinal inflammation, and altered nutrient metabolism in HZ patients, it suggests a close association between HZ development and immune system disturbances, particularly in elderly individuals with reduced immune function (34, 35). Immune system disruptions can alter the intestinal environment, potentially inhibiting the growth of the *Eubacterium hallii* group. Moreover, VZV infection may directly or indirectly impact the growth conditions for these probiotics. The altered intestinal microenvironment further challenges the maintenance of normal *Eubacterium hallii* group abundance. Inflammatory mediators such as TNF-α and IL-6 can compromise intestinal barrier function and disrupt microbiome balance (36). Additionally, HZ patients often experience altered nutrient metabolism, such as inadequate intake or malabsorption, which can affect intestinal flora composition. The *Eubacterium hallii* group relies heavily on specific nutrients like dietary fiber, and changes in nutrient availability may restrict its growth. Similar to inflammatory bowel disease (IBD) and metabolic syndrome, the *Eubacterium hallii* group exhibits reduced abundance in other diseases, indicating sensitivity to intestinal conditions and susceptibility to inflammation and nutritional status (37).

Decreased immune system function in patients with HZ may contribute to an increase in the abundance of Escherichia/Shigella spp., Veillonella spp., and phylum Proteobacteria. These conditionally pathogenic bacteria, including certain strains of Escherichia/Shigella and Proteobacteria, tend to proliferate in immunocompromised individuals. Escherichia/Shigella and Veillonella are particularly adaptable to inflammatory environments, utilizing metabolites produced during inflammation as nutrients, which promotes their rapid growth (38). For instance, Veillonella spp. can utilize lactic acid as a carbon source, giving them a competitive edge in inflammatory settings (39). These findings suggest that these microorganisms could have potentially contributed to inflammation and metabolic disorders.

Nevertheless, our study has several limitations. First, due to constraints in GWAS data, we could only investigate the relationship between genus-level or higher taxa and HZ, lacking the ability to validate specific species-level associations. Second, the analysis was constrained by the limited availability of extensive HZ datasets, potentially limiting the depth of our findings. There is also a possibility of false positives arising from multiple corrections. Moreover, mutations affecting other pathways could impact the validity of our results. Additionally, direct measurement of gut microbiota in tissue samples was not feasible. Further investigations using clinical samples are essential to establish a more precise correlation between transgenic levels and HZ, particularly in terms of specific microbiome increases or decreases. Furthermore, our study population consisted solely of individuals from European backgrounds; expanding geographically and ethnically would enhance the relevance of our findings for global HZ prevention and treatment efforts. Finally, potential confounding factors such as antibiotics, proton pump inhibitors (PPIs), and nonsteroidal anti-inflammatory drugs (NSAIDs) must be further refined. Antibiotic use may disrupt gut flora, reducing probiotic abundance, while immunosuppressive and antineoplastic drugs heighten shingles risk by suppressing immune function. Mental stress impacts the gut microbiome via the gut-brain axis, impairing barrier function and altering composition and function. Smoking and alcohol consumption also significantly influence the gut microbiome; smokers exhibit reduced flora diversity and increased pathogenic bacteria, while alcohol disrupts metabolic functions and increases gut permeability.

These findings represent a pioneering step forward, offering new avenues for addressing HZ as a significant global public health challenge. For instance, strategies targeting detrimental flora like Tyzzerella 3 and Cyanobacteria could potentially reduce HZ risk, achievable through dietary adjustments, probiotic supplementation, or other microbiome interventions. Secondly, modifying the intestinal environment to discourage harmful flora proliferation may alleviate HZ symptoms and complications. Promoting beneficial flora growth via dietary fiber-rich foods, for instance, can enhance gut health and lower inflammation levels, indirectly inhibiting VZV virus reactivation. Moreover, stress is a recognized HZ trigger. Techniques such as positive thinking, yoga, and relaxation practices can mitigate stress effects on the gut-brain axis, offering novel approaches to HZ prevention. Concurrently, global public health initiatives could explore regular monitoring of gut flora composition in vulnerable populations like the elderly and immunocompromised, potentially advancing HZ prevention efforts.

In conclusion, we analyzed the causal relationship between 211 gut microbes and HZ. We confirmed that 2 gut microbiota may act as risk factors for HZ, while 4 gut microbiota may be influenced by HZ conditions.

## Data Availability

Publicly available datasets were analyzed in this study. This data can be found at: GWAS (https://gwas.mrcieu.ac.uk/). The dataset is named finn-b-AB1_ZOSTER.

## Footnotes

^1^ https://gwas.mrcieu.ac.uk/

^2^ http://www.R-project.org

## References

[1] Parikh R Spence O’M Giannelos N Kaan I. Herpes zoster recurrence: a narrative review of the literature. Dermatol Ther. (2024) 14:569–92. doi: 10.1007/s13555-024-01101-7 PMC10965844 38416279

[2] Vijay A Valdes AM. Role of the gut microbiome in chronic diseases: a narrative review. Eur J Clin Nutr. (2021) 76:489–501. doi: 10.1038/s41430-021-00991-6 34584224 PMC8477631

[3] Deng Z Liu Y Wang H Luo T. Genetic insights into the gut microbiota, herpes zoster, and postherpetic neuralgia: a bidirectional two-sample Mendelian randomization study. Front Genet. (2024) 15:1366824. doi: 10.3389/fgene.2024.1366824 38846958 PMC11153692

[4] Burgess S Timpson NJ Ebrahim S Davey Smith G. Mendelian randomization: where are we now and where are we going? Int J Epidemiol. (2015) 44:379–88. doi: 10.1093/ije/dyv108 26085674

[5] Davies NM Holmes MV Davey Smith G. Reading Mendelian randomisation studies: a guide, glossary, and checklist for clinicians. BMJ. (2018) 362:k601. doi: 10.1136/bmj.k601 30002074 PMC6041728

[6] Liu X Zou L Nie C Qin Y Tong X Wang J. Mendelian randomization analyses reveal causal relationships between the human microbiome and longevity. Sci Rep. (2023) 13:31115. doi: 10.1038/s41598-023-31115-8 PMC10052271 36991009

[7] Li L Fu L Zhang L Feng Y. Varicella-zoster virus infection and primary membranous nephropathy: a Mendelian randomization study. Sci Rep. (2023) 13:19212. doi: 10.1038/s41598-023-46517-x 37932291 PMC10628161

[8] Zou M Zhang W Shen L Xu Y Zhu Y. Causal association between inflammatory bowel disease and herpes virus infections: a two-sample bidirectional Mendelian randomization study. Front Immunol. (2023) 14:1203707. doi: 10.3389/fimmu.2023.1203707 37465669 PMC10351388

[9] Liu X Li X Xie M Guo J Zheng X Shi S. Association of gut microbiome and oral cavity cancer: a two sample mendelian randomization and case-control study. J Stomatol Oral Maxillofac Surg. (2023) 125:101736. doi: 10.1016/j.jormas.2023.101736 38086473

[10] Kurilshikov A Medina-Gomez C Bacigalupe R Radjabzadeh D Wang J Demirkan A. Large-scale association analyses identify host factors influencing human gut microbiome composition. Nat Genet. (2021) 53:156–65. doi: 10.1038/s41588-020-00763-1 33462485 PMC8515199

[11] Kurki MI Karjalainen J Palta P Sipilä TP Kristiansson K Donner KM. FinnGen provides genetic insights from a well-phenotyped isolated population. Nature. (2023) 613:508–18. doi: 10.1038/s41586-022-05473-8 36653562 PMC9849126

[12] Cheng Q Zhang X Chen LS Liu J. Mendelian randomization accounting for complex correlated horizontal pleiotropy while elucidating shared genetic etiology. Nat Commun. (2022) 13:6490. doi: 10.1038/s41467-022-34164-1 36310177 PMC9618026

[13] Lin L Zhang R Huang H Zhu Y Li Y Dong X. Mendelian randomization with refined instrumental variables from genetic score improves accuracy and reduces Bias. Front Genet. (2021) 12:618829. doi: 10.3389/fgene.2021.618829 33868364 PMC8044958

[14] Yu H Wan X Yang M Xie J Xu K Wang J. A large-scale causal analysis of gut microbiota and delirium: a Mendelian randomization study. J Affect Disord. (2023) 329:64–71. doi: 10.1016/j.jad.2023.02.078 36842654

[15] Xu Q Ni JJ Han BX Yan SS Wei XT Feng GJ. Causal relationship between gut microbiota and autoimmune diseases: a two-sample Mendelian randomization study. Front Immunol. (2021) 12:746998. doi: 10.3389/fimmu.2021.746998 35140703 PMC8819003

[16] Thomas H. Mendelian randomization reveals causal effects of the gut microbiota. Nat Rev Gastroenterol Hepatol. (2019) 16:198–9. doi: 10.1038/s41575-019-0133-y 30850821

[17] Xiao Q-A Yang Y-F Chen L Xie Y-C Li H-T Zhi-Gang F. The causality between gut microbiome and liver cirrhosis: a bi-directional two-sample Mendelian randomization analysis. Front Microbiol. (2023) 14:1256874. doi: 10.3389/fmicb.2023.1256874 37920262 PMC10619669

[18] Zeng Y Cao S Yang H. Roles of gut microbiome in epilepsy risk: a Mendelian randomization study. Front Microbiol. (2023) 14:1115014. doi: 10.3389/fmicb.2023.1115014 36922970 PMC10010438

[19] Zhang Q Zhou J Zhang X Mao R Zhang C. Mendelian randomization supports causality between gut microbiota and chronic hepatitis B. Front Microbiol. (2023) 14:1243811. doi: 10.3389/fmicb.2023.1243811 37655340 PMC10467284

[20] Burgess S. Sample size and power calculations in Mendelian randomization with a single instrumental variable and a binary outcome. Int J Epidemiol. (2014) 43:922–9. doi: 10.1093/ije/dyu005 24608958 PMC4052137

[21] Cai Y Wen S Hu J Wang Z Huang G Zeng Q. Multiple reports on the causal relationship between various chronic pain and gut microbiota: a two-sample Mendelian randomization study. Front Neurosci. (2024) 18:1369996. doi: 10.3389/fnins.2024.1369996 38694896 PMC11061420

[22] Bowden J Davey Smith G Haycock PC Burgess S. Consistent estimation in Mendelian randomization with some invalid instruments using a weighted median estimator. Genet Epidemiol. (2016) 40:304–14. doi: 10.1002/gepi.21965 27061298 PMC4849733

[23] Burgess S Thompson SG. Interpreting findings from Mendelian randomization using the MR-egger method. Eur J Epidemiol. (2017) 32:377–89. doi: 10.1007/s10654-017-0255-x 28527048 PMC5506233

[24] Burgess S Foley CN Allara E Staley JR Howson JMM. A robust and efficient method for Mendelian randomization with hundreds of genetic variants. Nat Commun. (2020) 11:376. doi: 10.1038/s41467-019-14156-4 31953392 PMC6969055

[25] Verbanck M Chen CY Neale B Do R. Detection of widespread horizontal pleiotropy in causal relationships inferred from Mendelian randomization between complex traits and diseases. Nat Genet. (2018) 50:693–8. doi: 10.1038/s41588-018-0099-7 29686387 PMC6083837

[26] Han S Gao J Wang Z Xiao Y Ge Y Liang Y. Genetically supported causality between gut microbiota, immune cells and morphine tolerance: a two-sample Mendelian randomization study. Front Microbiol. (2024) 15:1343763. doi: 10.3389/fmicb.2024.1343763 38389539 PMC10882271

[27] Cohen JF Chalumeau M Cohen R Korevaar DA Khoshnood B Bossuyt PM. Cochran's Q test was useful to assess heterogeneity in likelihood ratios in studies of diagnostic accuracy. J Clin Epidemiol. (2015) 68:299–306. doi: 10.1016/j.jclinepi.2014.09.005 25441698

[28] Barili F Parolari A Kappetein PA Freemantle N. Statistical primer: heterogeneity, random-or fixed-effects model analyses? Interact Cardiovasc Thorac Surg. (2018) 27:317–21. doi: 10.1093/icvts/ivy163 29868857

[29] Zhang B Huang X Wang X Chen X Zheng C Shao W. Using a two-sample mendelian randomization analysis to explore the relationship between physical activity and Alzheimer's disease. Sci Rep. (2022) 12:12976. doi: 10.1038/s41598-022-17207-x 35902670 PMC9334579

[30] Li J Tang M Gao X Tian S Liu W. Mendelian randomization analyses explore the relationship between cathepsins and lung cancer. Commun Biol. (2023) 6:1019. doi: 10.1038/s42003-023-05408-7 37805623 PMC10560205

[31] Hamjane N Mechita MB Nourouti NG Barakat A. Gut microbiota dysbiosis-associated obesity and its involvement in cardiovascular diseases and type 2 diabetes. A systematic review. Microvasc Res. (2024) 151:104601. doi: 10.1016/j.mvr.2023.104601 37690507

[32] Zhu T Liu X Yang P Ma Y Gao P Gao J. The association between the gut microbiota and erectile dysfunction. World J Mens Health. (2024). doi: 10.5534/wjmh.230181 PMC11439808 38311371

[33] Mello FD Braidy N Marçal H Guillemin G Nabavi SM Neilan BA. Mechanisms and effects posed by neurotoxic products of Cyanobacteria/microbial eukaryotes/dinoflagellates in algae blooms: a review. Neurotox Res. (2018) 33:153–67. doi: 10.1007/s12640-017-9780-3 28836116

[34] Mann ER Lam YK Uhlig HH. Short-chain fatty acids: linking diet, the microbiome and immunity. Nat Rev Immunol. (2024). doi: 10.1038/s41577-024-01014-8 38565643

[35] Park J Kim CH. Regulation of common neurological disorders by gut microbial metabolites. Exp Mol Med. (2021) 53:1821–33. doi: 10.1038/s12276-021-00703-x 34857900 PMC8741890

[36] Haroun E Kumar PA Saba L Kassab J Ghimire K Dutta D. Intestinal barrier functions in hematologic and oncologic diseases. J Transl Med. (2023) 21:233. doi: 10.1186/s12967-023-04091-w 37004099 PMC10064590

[37] Wu K Luo Q Liu Y Li A Xia D Sun X. Causal relationship between gut microbiota and gastrointestinal diseases: a mendelian randomization study. J Transl Med. (2024) 22:92. doi: 10.1186/s12967-024-04894-5 38263233 PMC10804519

[38] Zhao M Chu J Feng S Guo C Xue B He K. Immunological mechanisms of inflammatory diseases caused by gut microbiota dysbiosis: a review. Biomed Pharmacother. (2023) 164:114985. doi: 10.1016/j.biopha.2023.114985 37311282

[39] Zhou P Manoil D Belibasakis GN Kotsakis GA. Veillonellae: beyond bridging species in Oral biofilm ecology. Front Oral Health. (2021) 2:774115. doi: 10.3389/froh.2021.774115 35048073 PMC8757872

